# Genetic Evolution and Biological Characteristics of Feline Caliciviruses Isolated from Dogs

**DOI:** 10.1155/2023/1145176

**Published:** 2023-02-27

**Authors:** Fanyuan Sun, Xinyi Guo, Jinfan Guo, Min Zhu, Huabo Zhou, Jiancai Chen, Xin Huang, Hewei Chen, Yi Xu, Yaohui Zhu, Pingping Wang, Chongqiang Huang, Jianming Long, Kang Ouyang, Zuzhang Wei, Weijian Huang, Ying Chen

**Affiliations:** ^1^Laboratory of Animal Infectious Diseases and Molecular Immunology, College of Animal Science and Technology, Guangxi University, Nanning 530004, China; ^2^Huabo Pet Hospital, Nanning 530004, China; ^3^Shenjiu Biological Products Co., Ltd., Nanning 530004, China; ^4^Guangxi Zhuang Autonomous Region Engineering Research Center of Veterinary Biologics, Nanning 530004, China; ^5^Guangxi Key Laboratory of Animal Reproduction, Breeding and Disease Control, Nanning 530004, China; ^6^Guangxi College and Universities Key Laboratory of Prevention and Control for Animal Disease, Nanning 530004, China

## Abstract

Feline calicivirus (FCV) is a highly contagious pathogen associated with oral and upper respiratory tract diseases (URTD), and it is also possibly considered as an enteric pathogen. Some studies found FCV-like viruses in the enteric tract of dogs, but there was a lack of understanding regarding the epidemiology and biological properties of FCVs in dogs. In this study, 252 fecal/feces samples were collected from dogs, with or without diarrhea, from 2020 to 2021. There were 6 FCV-positive samples (2.41%, 6/252), from which only two FCVs were successfully isolated and the complete genome sequences obtained. Phylogenetic analysis showed that the two canine-origin FCV isolates belonged to genogroup I and formed a monophyletic cluster with previous FCV strains, sharing a common ancestor. However, there was genetic diversity when the nt identity of the VP1 proteins between the two canine-origin FCV isolates (77.4% nt identity) was compared. In particular, the genomic sequence of the canine/GXHC01-21 isolate showed evidence of recombination at the 3ʹ end of the ORF1 gene with sequence identity very similar to the FCV strain, GX2019, previously isolated from cats in Guangxi in 2019. A comparison of their replication properties indicated that the two isolates could not replicate efficiently in MDCK cells. This was also seen in the enteric FCV isolate, GXNN04-20. However, both displayed similar plaque phenotypes to the respiratory FCV isolate, GX01-13. In addition, it was found that sera from vaccinated cats had low cross-reactivity in a neutralizing antibody test against the two canine-origin FCV isolates. Moreover, high neutralizing antibody titers (≥1 : 128) against canine-origin FCV viruses were observed in the two canine serum samples. This confirmed that interspecies transmission had occurred between cats and dogs. Our results provided an in-depth understanding of the genetic evolution and characteristics of FCVs circulating in dogs.

## 1. Introduction

Feline calicivirus (FCV) has a single-stranded, positive-sense genomic RNA of ∼7.7 kb organized into three major open-reading frames (ORFs). The 5ʹ untranslated region (UTR) of the genome is linked covalently by the VPg protein while the 3ʹ UTR terminates with a poly (A) [1–3]. ORF1 encodes a polyprotein that is posttranslationally processed into several nonstructural proteins [2]. ORF2 encodes the major capsid protein (VP1), which is divided into six regions, A–F, based on their sequence conservation [4]. The hypervariable region, E, is thought to map the neutralizing epitopes and the cellular receptor (feline junctional adhesion molecule 1, fJAM-1), as well as its immune recognition by the host [5–9]. ORF3 encodes for the minor capsid protein (VP2), which is essential for the production of infectious virions, and it facilitates the delivery of the FCV genome into the host cell by the formation of channel-like structures [10, 11].

FCV is one of the important viral pathogens of upper respiratory tract disease (URTD) in domestic cats worldwide. Ulcerative disease, gingivitis-stomatitis, and limping syndrome are also FCV-specific diseases [12]. The disease manifestation depends on host immunity, the multicat environment, and coinfection. More recently, the emergence of highly virulent strains of FCVs (VS-FCVs) has been reported in China [13, 14] and Europe [15, 16]. These may cause multiorgan infections, persistent fever, edema of the face and limbs, pancreatitis, and hepatic necrosis in cats. Also, FCVs have constantly been detected from the enteric tracts of cats, and this could be an important for the evolution of FCVs [17, 18].

FCV belongs to the genus *Vesivirus* and family *Caliciviridae.* This family includes eleven genera, and these are *Bavovirus, Lagovirus, Minovirus, Nacovirus, Norovirus, Nebovirus, Recovir*us, *Salovirus, Sapovirus, Valovirus,* and *Vesivirus*. They can infect human and a wide range of animals [19]. Several caliciviruses have been identified in dogs, and they show phylogenetic relationships to the *Vesiviruses* genus, but they differed genetically and antigenically from FCVs [20–22]. Moreover, FCV-like particles have been also isolated from diarrhea samples of dogs [23–25]. These were clustered with the FCV group, and the animals showed a high prevalence of antibodies against FCV-F9 (the vaccine strain), suggesting that there is a circulation of FCVs among dogs and cats [23]. However, data regarding the genomic characterization and growth properties of FCV-like particles in dogs are still unclear. Here, we isolated two FCV strains identified in canines. In this study, the genetic evolution and phenotypic characterization of two canine-origin FCVs were demonstrated. This will increase our knowledge towards understanding the interspecies circulation of FCVs between cats and dogs.

## 2. Materials and Methods

### 2.1. Sample Collection

In 2021, 252 fecal/feces samples were collected from dogs with diarrhea and vomiting (*n* = 129) or those without diarrhea (*n* = 123). From these, six FCV-positive samples were identified by RT-PCR. The detailed clinical information of these FCV-positive dogs, including the dog breed, locations, ages, sex, and clinical signs as well as the origin and collection dates of the samples and name of the isolate, is shown in Table 1.

### 2.2. Screening by RT-PCR

Viral RNA was extracted from either fecal swabs or 20% (w/v) feces by using an AxyPrep Body Fluid Viral DNA/RNA Miniprep Kit (AXYGEN, USA) and following the manufacturer's instructions. cDNA was generated by using reverse transcriptase M-MLV (Takara Bio, Inc., Dalian, China) and oligo (dT) primers. The samples were screened using RT-PCR with 28 F (5ʹ-AACTCTGAGCTTCGTGCT-3ʹ) and 413 R (5ʹ-TDAGCTGTTCTTTRCACA -3ʹ) primer pairs, which targeted a 386-bp length of the ORF1 gene. This is a conserved region as judged by the alignment of 60 FCV complete genomic sequences available from NCBI. The cDNA templates were denatured at 95°C for 5 min, followed by 30 cycles of denaturation at 95°C for 30 s, annealing at 45°C for 30 s and extension at 72°C for 30 s, and final extension at 72°C for 10 min. The PCR products were analyzed by agarose gel electrophoresis, and these were stained with ethidium bromide and then visualized under UV light.

### 2.3. Virologic Investigation

As described previously, the FCV-positive samples were processed and inoculated onto confluent Crandell-Reese feline Kidney (CRFK) cells until the obvious cytopathic effects (CPEs) were observed [18]. The cell supernatants were then used for the amplification of the complete FCV genome. The infected cells were tested for FCVs by using the indirect immunofluorescence assay (IFA). CRFK cells were inoculated at a multiplicity of infection (MOI) of 0.0001, and cells were observed for CPEs after 24 h.p.i. Cells were then fixed and subjected to indirect immunofluorescence. The primary antibody used was derived from positive serum obtained from cats challenged with the GX01-13 strain. The secondary antibody was a commercial FITC-goat anti-cat IgG. The nuclei were stained with DAPI.

### 2.4. Amplification of the Complete Genome

According to previous protocols, the complete genome was divided into four overlapping fragments, and four sets of primers were employed to amplify these [18]. The reaction of PCR was predegenerated at 98°C for 2 min, 34 cycles of denaturation at 98°C for 15 s, annealing at 48°C for 15 s, and extension at 72°C for 90 s, followed by a final extension at 72°C for 8 min. The PCR products were purified, and these were directly sequenced by using Sanger's method (Sangon Bio, Inc., Guangzhou, China).

### 2.5. Sequence Analysis

Two complete genome sequences were aligned and analyzed using the SeqMan and MegAlign programs (DNASTAR, Madison, USA), respectively. These were then analyzed using the BLAST program in NCBI (http://blast.ncbi.nlm.nih.gov/Blast.cgi). The nucleotide sections of the genome sequences and the ORF2 genes were aligned with published FCVs reference strains by using ClustalW included in MEGA 11.0 (http://www.megasoftware.net/). The reference sequences included 11 previously isolated FCV strains obtained in Guangxi as well as 33 FCV genomic and 53 capsid sequences available in the GenBank database. The same software packages were used to construct phylogenetic trees by using the maximum likelihood (ML) method with the Tamura-Nei and Kimura 2-parameter models, respectively. These were evaluated by using the bootstrap test value of 1000 replicates. The putative recombinant origin of the two canine-origin isolates was assessed using a recombination detection program (RDP5.3) software package [26]. The alignment of the nucleotide sequences for their complete genomes was analyzed using the SimPlot v3.5.1 software. The phylogenetic trees based on each recombinant fragment were constructed to display the accuracy of recombinant events.

### 2.6. Evaluation of Susceptibility of Viruses to pH, Trypsin, and Bile Salts

The two FCV isolates from dogs (canine/GXHC01-21 and canine/GXNN10-21) were compared with enteric FCV (E-FCV), GXNN04-20, and the respiratory FCV (R-FCV), GX01-13 isolates from cats under the conditions of low pH, trypsin, and bile salts *in vitro* in accordance with Di Martino's protocol [17]. Hydrochloric acid was added to the virus suspension to reduce its final pH to 3.0. The virus suspensions were also treated with 1% trypsin. Some samples were mixed with an equal volume of bile salt mixture (Sigma-Aldrich) at a final concentration of 0.5% and then incubated for 1 h at 37°C in a 5% CO_2_ incubator. The viral titers were then evaluated by the Reed and Muench method, as described previously [18].

### 2.7. Plaque Assay

Monolayers of CRFK cells were cultured, and tenfold serial dilutions of virus suspensions were inoculated into these. After adsorption for 1 h at 37°C with gentle agitation every 10 min, the cells were washed three times with PBS and overlaid with 3 mL of an agar medium containing 2 × MEM (Gibco, USA) media, 1% (w/v) DEAE Dextran (Sigma Aldrich, USA), 5% NaHCO_3_ (Sigma), and 1.8% (w/v) purified agar (Oxoid, UK). After incubation at 37°C for 36 h, the overlay was removed, and plaques were visualized by staining with 0.05% (w/v) crystal violet solution. The individual plaque areas were measured using the Image J software.

### 2.8. Replication Properties in MDCK and CRFK Cells

CRFK and MDCK cells were grown to confluence in 24-well tissue culture plates and inoculated with the two canine-origin FCV isolates, respiratory FCV (R-FCV) strain GX01-13, and enteric FCV (E-FCV) strain GXNN04-20, at a MOI of 5, respectively. After 1 h of adsorption at 37°C in an atmosphere of 5% CO_2_, the cells were washed twice with phosphate-buffered saline (PBS). Dulbecco's modified Eagle's medium (DMEM) supplemented with 4% FBS (Gibco, USA) was then added. The cell supernatants were collected at 1, 2, 4, 8, 12, 18, 24, 36, and 48 hours postinfection. Three replicate wells were used for each time point. The culture supernatants were collected at various times, and these were frozen at −80°C for the subsequent determination of their TCID_50_.

### 2.9. Antibody Neutralizing Assay

One hundred serum samples from dogs (*n* = 50) and cats (*n* = 50) with known vaccination histories were collected and tested for neutralizing antibodies against the canine-origin isolates, canine/GXNN10-21 and canine/GXHC01-21. The serum samples were collected from cats with a known prior vaccination history. Five hundred *μ*l of serum samples were inactivated at 56°C for 30 min and filtered through 0.22 *μ*M Millipore filters (Bedford, MA). Fifty *μ*l of serially twofold diluted serum and 50 *μ*l of infectious medium with 100 TCID_50_ of canine/GXHC01-21 and canine/GXNN10-21 viruses were mixed and added to 96-well plates for incubation at 37°C for 1 h. The appropriate dilutions of the mixture were inoculated into monolayers of CRFK cells at 37°C in an atmosphere of 5% CO_2_ for 5 days. Four replicates were used for each dilution per serum sample. DMEM without FBS was used as a negative control, and FCV-positive sera were collected when cats were challenged by FCV GX01-13 virus at 14 days postinfection. Antibody titers were determined by assessing the highest dilution of the serum sample used, which resulted in 50% of the CRFK cells showing no CPE. The neutralization titer was calculated by using the Reed-Muench method.

## 3. Results

### 3.1. Virus Isolation and Identification

**Figure 1:**
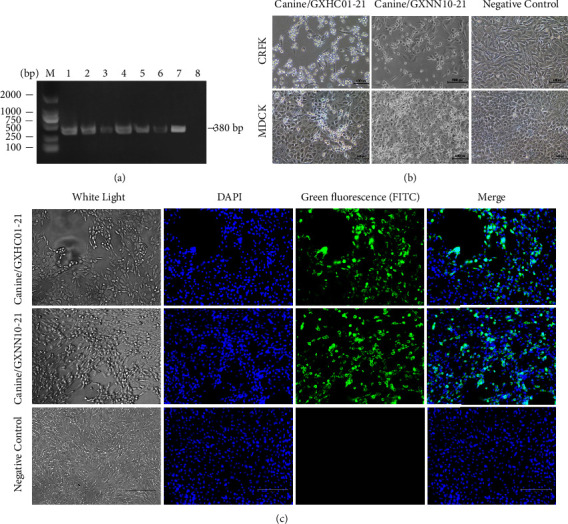
Isolation and identification of two FCV strains from dogs. (a) RT-PCR with 28F/413R primer sets. M: Marker (Takara DNA Ladder 2000); Line 1–6: PCR products of six FCV-positive samples from dogs of 380 bp in size obtained using feline calicivirus primers. Line 7: positive control. Line 8: negative control. (b) Cytopathic effects (CPEs) of two canine-origin FCV isolates on CRFK and MDCK cells. CRFK and MDCK cells were inoculated with two isolates (canine/GXHC01–21 and canine/GXNN10-21) and negative medium, as shown at 12 hours post-infection. Magnification, 200×. (c) Indirect immunofluorescence analysis on CRFK cells. CRFK cells were inoculated with the two strains of canine viruses at a multiplicity of infection of 0.01 and observed at 12 hours post-infection. The green fluorescence on CRFK cells was revealed by using a cat polyclonal serum against the FCV GX01-13 strain. The CPE were observed using white light and the nuclei strained by DAPI are shown as blue.

**Figure 2:**
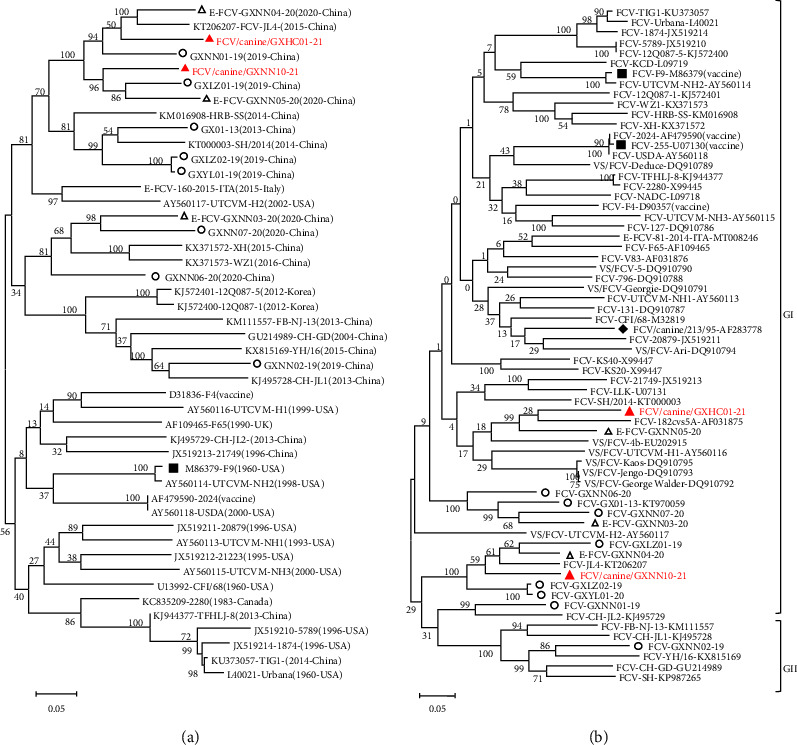
Phylogenetic trees of two canine-origin FCV isolates, 11 previous FCV isolates and referenced strains available from GenBank based on the complete genome (a) and the nucleotides sequences of the ORF2 genes (b). Phylogenetic trees were generated by using the maximum likelihood (ML) method with the Tamura-Nei (a) and Kimura 2-parameter (b) models. *A* value ≥ 70 for the ML bootstrap (1000 replications) is shown at the main branch of the trees. The two canine-origin isolates obtained in this study are marked with “red triangles”; The FCV strain canine/213/95 isolated from Italy [24] is indicated with a “black diamond”; The commonly used vaccine strains are indicated with “black squares”; Previous published FCV isolates from the respiratory and enteric tracts are marked with “hollow circles and triangles”, respectively.

In 2021, there were six FCV-positive fecal samples detected by RT-PCR by using primers 28F and 418R specific for FCVs (Figure 1). These included five FCV-positive samples from dogs with diarrhea that were also coinfected with either canine parvovirus (CPV), canine coronavirus (CCoV), or canine distemper virus (CDV). There was also a case from a healthy dog. Among these samples, two FCV-like viruses were successfully isolated from fecal/feces samples by inoculating onto CRFK cells, named as canine/GXHC01-21 and canine/GXNN10-21 (Table 1). At 24 hours postinfection, the infected cells exhibited obvious cell rounding and cluster-like structures. In contrast, the CPEs produced on MDCK cells seemed to be less potent than those seen with CRFK cells, and they were rounded and exfoliated. The control group did not show any cytopathy (Figure 1(b)). The isolated viruses were identified by using anti-FCV cat serum and FITC-conjugated AffiniPure goat anti-cat IgG (Figure 2).

### 3.2. Sequence Analysis

The sequenced genome sequences of the two canine-originated FCV isolates were 7721 and 7722 nucleotides long for canine/GXHC01-21 and canine/GXNN10-21, respectively. The 5ʹUTRs of these two isolates were conserved, but there was a small amount of nucleotide variations in the 3ʹUTRs (Table S1). Comparative analysis of the complete genome sequences with previous FCV isolates from Guangxi showed that their sequence identities ranged from 79.5 to 85.6%, compared with the previous FCV isolates of enteric and respiratory origins. The nt identity relative to the vaccine strain 255 was of average 79% (Table 2).

The sequence of 5292 nucleotides in the ORF1 gene encodes an RNA-dependent RNA polymerase (RdRp) with a predicted protein of 1763 aa. The two canine-originated FCV isolates shared 92.0–96.7% aa identities with previous FCV isolates and the vaccine strain 255. Compared with the previous FCV isolates (including those of canine origin), the results showed that their sequence identities with respect to the ORF2 nucleotide sequences ranged from 75.2 (canine/213/95) to 84.2% (R-FCV GXNN01-19). The corresponding amino acid sequences varied from 85.4 to 93.1%. Sequence identities of the region E between the two isolates were as low as 74.7% at the amino acid level, and with those of other FCV isolates, it ranged from 74.5 (E-FCV GXNN05-20) to 86.7% (R-FCV GXNN01-19). Interestingly, the two canine-originated FCV isolates in this study showed lower identities of 64.2–69.7%, when compared with the FCV-like viruses from dogs in Italy.

**Figure 3:**
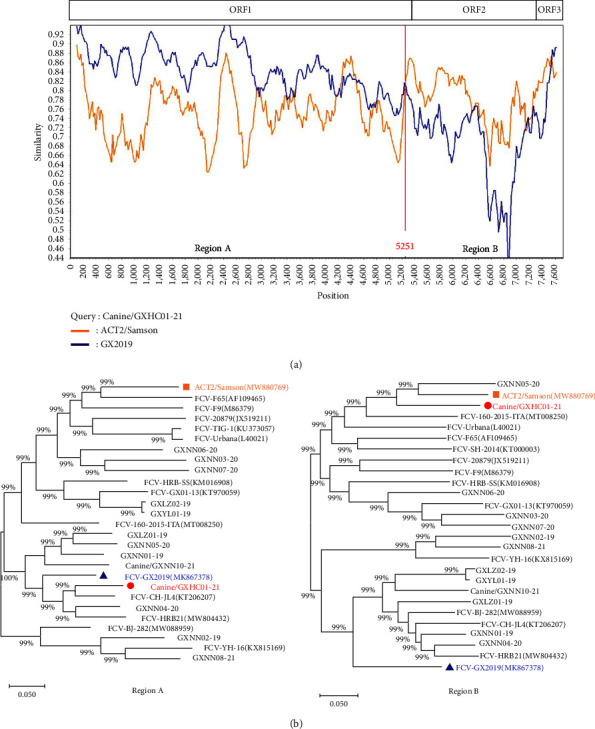
The complete genome recombination analysis of strain canine/GXHC01-21. (a) Comparison of the genomic similarity of strain canine/GXHC01-21 (query) with R-FCV GX2019 (blue) and ACT2/Samson (orange) using a sliding window. The potential recombination breakpoints are shown as red vertical lines and they are marked with nucleotide sites at the bottom of window; (b) Genetic evolution trees based on every recombinant fragment within canine/GXHC01-21 and the 26 FCV reference strains are shown below the similarity plot. The strain canine/GXHC01-21 is marked with a “red circle”, the major parental strain, GX2019, is marked with a “blue triangle”, and the minor parental strain, ACT2/Samson, is marked with an “orange square”.

Recombination events were thought to drive genetic evolution and produce novel viruses. To examine whether there were possible recombinant events between the two canine-originated FCV isolates, 26 FCV genomic sequences were analyzed using the software packages available in Simplot and RDP5.3 (Figure 3). The results showed that the canine/GXHC01-21 genomic sequence had the evidence of recombination with scores of 0.626. The putative recombination event was detected by six methods (RDP 1.777 × 10^−9^, BootScan 4.842 × 10^−7^, MaxChi 2.396 × 10^−18^, Chimaera 5.753 × 10^−4^, SiScan 7.640 × 10^−13^, and 3Seq 9.972 × 10^−9^). The recombination breakpoints were mapped to position 5251 of the genomic sequence (the 3ʹ end of ORF1 gene). The major parent strain was considered to involve the FCV-GX2019 strain isolated from cats in Guangxi in 2019 (86.4% similarity). The minor parent strain was genetically related to ACT2/Samson strain obtained from cats with URTD [26] (Figure 3(b)).

### 3.3. Phylogenetic Analysis

To determine the genetic characterization of the two canine-originated FCV isolates, two phylogenetic trees based on the genomic sequences and capsid proteins were constructed (Figure 2). Phylogenetic analysis based on the complete genome sequences showed that the two isolates were clustered with the E-FCV strains (GXNN04-20 and GXNN05-20) and R-FCV strains (GXNN01-19 and GXLZ01-19). They were mostly related to strain CH-JL4 which was isolated from the North of China in 2015 and formed a small branch, sharing 82.2–85.3% nt identities (Figure 2(a)). Comparative analysis of the capsid sequences from 66 FCV strains revealed that the two canine-originated isolates were divided into different clusters. The canine/GXHC01-21 isolate shared a close genetic relationship with the E-FCV strain (GXNN05-20) and five VS-FCV strains. In contrast, the strain canine/GXNN10-21 was closely related to the JL4-like FCV group, sharing high aa identities of 82.0–84.2% (Figure 2(b)). These results suggested that dogs can acquire FCV infection from cats and that they could also be a potential source of FCV infection.

### 3.4. Stability to Acid, Trypsin, and Bile Salts *in vitro*

The viral titers of the four different FCV strains were tested by their treatment with acid, trypsin, and bile salts (Table 3). The results showed that the canine/GXHC01-21 isolate lost most of its infectivity at low pH in a similar manner to the E-FCV strain (GXNN04-20). However, the canine/GXNN10-21 isolate was not susceptible to low pH and only showed a small loss of its infectious titer when compared to the other FCV viruses. In contrast, there was a limited reduction in the TCID_50_ for the two canine-originated isolates with respect to their sensitivities to trypsin. Their TCID_50_ varied from 1.23 log_10_ to 2.73 log_10_. Under bile salts conditions, the titer of strain canine/GXNN10-21 decreased by 1.83 log_10_, whilst the log_10_ reduction for canine/GXHC01-21 virus was 0.39. The two canine-originated FCV isolates exhibited a marked relative difference with respect to both low pH and bile salt conditions.

### 3.5. Replication on CRFK and MDCK Cells

**Figure 4:**
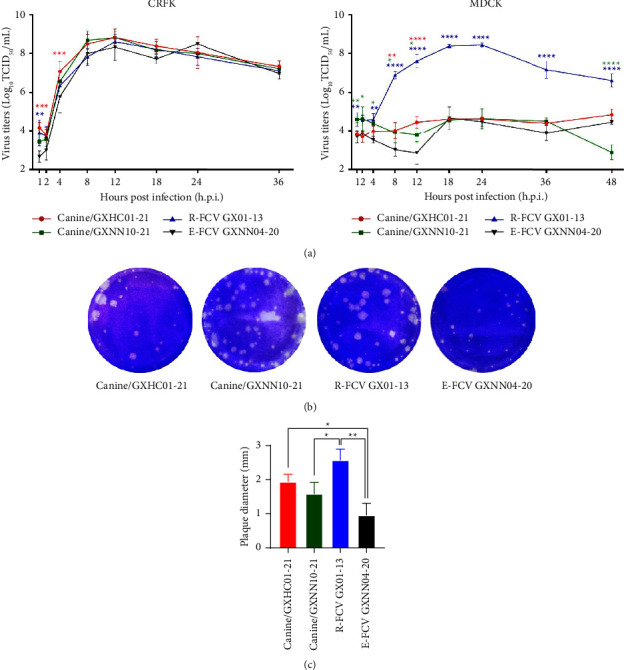
Comparative growth properties between the two canine-origin FCV isolates, R-FCV (GX01-13) and E-FCV (GXNN04-20). (a) One-step growth curves of viruses in CRFK and MDCK cells at a multiplicity of infection of 5 (^∗^*P* < 0.05; ^∗∗^*P* < 0.01; ^∗∗∗^*P* < 0.001; ^∗∗∗∗^*P* < 0.0001). (b) Plaque phenotype in monolayer CRFK cells were inoculated with 10-fold serial dilutions of each FCV strain at 37°C for 1 h. Infected cells were incubated for 36 hours post-infection, before staining with 0.8% (w/v) crystal violet solution. (c) The average plaque areas for the four FCV strains were measured and calculated using ImageJ software. Statistical analysis was performed by three-way ANOVA.

In order to understand whether the two canine-originated FCV isolates changed their cell-tropism properties, we determined their impacts on replication *in vitro* in canine (MDCK) and feline (CRFK) cell lines. Growth yields of the two canine-originated FCV isolates were similar to R-FCV GX01-13 and E-FCV GXNN04-21 in CRFK cells with an increased trend by 2–12 hours postinfection. In contrast, these strains showed significant replicative differences in MDCK cells. The GX01-13 strain could efficiently replicate in the MDCK cell cultures with a maximum yield (8.5 Log_10_ TCID_50_) observed at 24 hours postinfection. However, the two canine-originated isolates produced lower yields in MDCK cells, with similar growth properties to the E-FCV strain, GXNN04-20 (Figure 4). However, we found that the plaque sizes of the two canine-originated FCV isolates were similar to the R-FCV GX01-13 strain, which formed larger plaques. The E-FCV strain from cats formed the smallest plaques among these strains, suggesting the existence of different replicative phenotypes between the respiratory and enteric FCV viruses (Figure 4(b)).

### 3.6. Serological Investigation in Cats and Dogs

**Figure 5:**
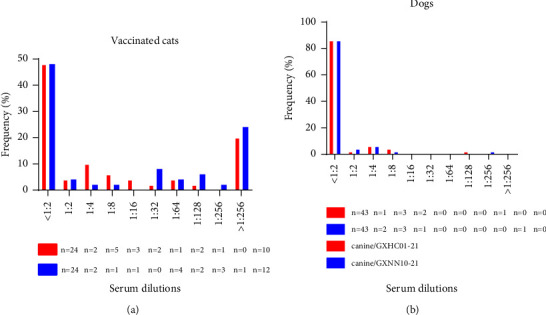
Distribution of neutralizing antibody titers against the two canine-origin FCV isolates in dogs and vaccinated cats. Fifty canine and fifty feline serum samples with known vaccination histories were collected. The neutralizing titers were determined by using a specific virus dilution in order to assess their 50% serum neutralization endpoint.

When the antibody titers in the vaccinated cats were >1 : 32, this was regarded as the protective neutralizing titer [27]. In our study, the prevalence of neutralizing antibodies in vaccinated cats with higher titers (>1 : 32) was only 28% (14/50) and 44% (22/50) when animal sera were tested using strains canine/GXHC01-21 and canine/GXNN10-21, respectively. Most of the cat sera (72% and 56%, for the 2 virus strains, respectively) presented with titers at lower dilutions (<1 : 16). This indicated that there was a poor antigenic cross-reactivity between the antibodies produced by the commercial vaccine and these two canine-originated FCV viruses. In contrast, the serum samples collected from dogs contained neutralizing titers with a prevalence of 14% (7/50)/14% (7/50), specific for strains canine/GXHC01-21 and canine/GXNN10-21, respectively. Notably, two of the dog sera obtained against the two canine-originated FCV viruses had higher titers (≥1 : 128). This strongly suggests that there is the potential for interspecies infection between cats and dogs (Figure 5).

## 4. Discussion

FCV is generally considered to be an important pathogen, and it is widespread in the general feline population, but it has been isolated from wild felids, indicating that there is a wide host range for the FCV infections [14, 28, 29]. Interestingly, it was found that there are occasional infections of FCV-like viruses in dogs with enteric symptoms [23–25, 30]. Herein, two FCV-like isolates from two-month-old puppies were found, and the animals were coinfected with other canine infectious viruses. Therefore, biological studies of the respiratory and enteric FCV strains obtained from feline and canine viruses were undertaken. This will give us an interesting insight into the ecology of FCVs among dogs and cats.

Comparative analysis of the complete genomes based on the phylogenetic trees revealed that the two canine-originated FCV isolates were grouped together with the previous FCV isolates of respiratory and enteric origins obtained in the Guangxi province, which is located in the south of China (Figure 2). This suggested that there was a genetic association between the geographical distribution and the clustering observed in the phylogenetic tree, which could be traced back to the early FCV strain (CH-JL4) from Northern China. The nt identity within the subgroup was 79.5–85.6%, whilst the two canine isolates were obtained from a separate city of Guangxi, and these were found to be more distantly related (Figure 2). In contrast, there was a marked genetic diversity between two canine isolates based on the complete capsid sequences.

Sequences analyses showed that the strain canine/GXNN10-21 was likely more antigenically related to some previously detected FCV strains, but strain canine/GXHC01-21 was clustered with the VS-FCV strains and one E-FCV strain (GXNN05-20). It showed genetic differences with the nt/aa identities of 77.4/86.7% when compared to strain canine/GXNN10-21. Notably, this strain had potential recombinant events with the previously isolated FCV strain GX2019 in Guangxi. This suggests that although the GX2019 strain was not the parental strain, there is likely to be some genetic link between this one and the newly found strain. RNA recombination events are considered to be the primary driving force of FCV evolution, and they can result in the production of novel variants. These were found in several FCV strains [26, 31–33], but the recombinant events of the FCV-like strains in the dog were the first to be reported in the study. Ludwig-Begall et al. assumed that possible interspecies recombination occurred in the feline Norovirus M49 strain [34, 35], suggesting the recombination was involved in the changes of adaptation to the new host. Whether the occurrence of recombinant events of FCVs in dogs will be considered as the primary impetus in their evolution and adaptation to dogs still needs continuous surveillance, and more genomic sequencing data need to be obtained before this can be ascertained.

Based on their stability to bile salts, it has been suggested that FCVs could be divided into respiratory (R) and enteric (E) types [36]. Some studies indicated that enteric FCVs were resistant to bile acids when compared to the respiratory FCVs. However, there are marked differences observed when trypsin is added to enteric and respiratory FCVs, and there were no significant titers when the newly found isolates were incubated with bile salts and at low pH conditions [17]. This is broadly in agreement with our previous studies [18]. In this study, we also found that the two canine viruses did not exhibit significant differences when compared to R-FCV and E-FCV with respect to bile salts, low pH conditions, and trypsin treatment. Moreover, more evidence is needed to determine whether these were spillover infections from E-FCV-infected cats. It could be that the FCVs were the primary causative agents of intestinal disease in dogs, which produced synergistic effects in coinfections with other enteric pathogens in the canine population.

FCVs showed restricted tropism for feline cells mediated by fJAM-1, which has been identified as a functional receptor for these viruses [8]. However, many studies have highlighted the importance of interaction with cell surface glycans, such as the observation that FCVs can also bind to *α* 2, 6-sialic acid on the surfaces of MDCK cells [37]. We also isolated the FCV strain (GX01-13) by inoculating the viruses into cultures of MDCK cells [38]. In order to evaluate whether the two canine-originated FCVs would prefer to bind to canine cell lines (MDCK), their replication kinetics were assessed in both two cell lines (MDCK and CRFK). The results showed that although these two canine viruses did not show increased cell tropism for MDCK cells, they did produce larger plaque sizes than that of the E-FCV strains (*P* < 0.05). Surprisingly, the GX01-13 strain isolated from MDCK cells produced their maximal yield in MDCK cell with a viral titer of 8.5 Log_10_. The TCID_50_ at 24 hours postinfection was similar when compared to the canine-originated and enteric FCV viruses. This strain not only underwent replacements in the genomic region but also caused acute respiratory and severe ulceration in the plantar regions after they were challenged in cats [18]. In common with FCVs, sialic acid can also be used as a coreceptor in reoviruses. The sialic-acid-binding reovirus strains altered the reovirus-host interaction, resulting in biliary disease [39]. Whether the repeated spillover infections of FCVs from cats to dogs will enhance their adaptation to the canine population and cause enteric disease in dogs still needs to be further investigated.

The seroprevalence specific for FCV-F9 in the canine population reached 63.9% [25], but there was a lower prevalence of 8.13% against the FCV strain, Te/10/07, isolated from dogs [23]. In the present study, there was a higher average prevalence of 15% in dogs against the FCV-like virus. Of note, there were two dog serum samples which contained neutralizing antibodies with higher titers (≥1 : 128), and this provided more evidence for the continued circulation of FCVs in dogs.

Immune-mediated positive selection is an important factor for generating the genetic diversity of FCVs. The emergence of accumulated mutations and recombination under the natural environment also accelerates the evolution of FCVs, which might result in an outbreak of VS-FCV and a shift of host. In our study, we confirmed that there is cross-species transmission between cats and dogs. This raises the concern that dogs may be a potential resource for the maintenance of FCV infection between cats and dogs.

## Figures and Tables

**Table 1:** Details of collection dates, locations, clinical signs of FCV-positive samples from dogs.

Dog breed	Date	Location	Age (months)	Sex	Clinical signs and pathogen diagnosis	Samples	Isolation	Access number in GenBank
Chinese garden dog	08/16/2021	Hechi	2	F	38.8°C, anorexia, depression, purulent discharge in the eyes, CCoV and CPV positive	Fecal	Canine/GXHC01-21	OP485690
Belgian malinois	10/10/2021	Nanning	2	F	Coughing, runing nose, sneezing, vomiting, depression, CDV and CCoV positive	Feces	Canine/GXNN10-21	OP485691
Labrador retrieve	11/16/2021	Hechi	23	F	Healthy	Feces	N/A	N/A
Bichon fries	12/30/2021	Nanning	24	F	Recovery after infection by CDV	Feces	N/A	N/A
Poodle	01/04/2022	Nanning	17	M	Fever, lower appetite, CDV positive	Feces	N/A	N/A
Chinese garden dog	01/07/2022	Nanning	3	F	Fever, bloody stools, CPV positive	Feces	N/A	N/A

^a^N/A, not applicable; URTD, upper respiratory tract disease; F, female; M, male; CCoV, canine coronavirus; CPV, canine parvovirus; CDV, canine distemper virus.

**Table 2:** Comparative analysis of genomic sequences of two canine-origin FCV isolates.

Origin	Strain	Canine/GXHC01-21 (nucleotide/amino acid)					Canine/GXNN10-21 (nucleotide/amino acid)				
		Genome	ORF1	ORF2	VP1-E^c^	ORF3	Genome	ORF1	ORF2	VP1-E^c^	ORF3
Canine	GXNN10-21	81.8	83.3/94.4	77.4/86.7	74.7	83.8/93.5	N/A	N/A	N/A	N/A	N/A
Feline	R-GX01-13	80.3	80.7/92.9	79.1/87.3	74.5	81.3/86.9	79.5	80.4/92.0	77.1/86.1	72.4	78.5/89.7
Feline	R-GXLZ01-19	82.1	84.0/95.4	77.1/87.7	80.6	82.9/91.6	83.9	85.9/95.0	82.0/92.4	73.5	88.8/98.1
Feline	R-GXNN01-19	82.5	84.6/95.1	77.3/88.1	77.6	81.3/88.8	85.6	86.0/95.7	84.2/93.1	86.7	88.8/95.3
Feline	E-GXNN04-20	84.3	87.2/96.7	77.0/87.3	77.6	82.9/92.5	83.9	83.7/93.9	84.0/92.8	82.7	88.8/99.1
Feline	E-GXNN05-20	83.1	83.8/95.0	80.4/89.4	73.5	87.2/95.3	83.5	86.2/95.7	76.1/87.0	74.5	83.5/95.3
Canine^a^	213/95	N/A	N/A	77.5/85.8	68.7	N/A	N/A	N/A	75.2/85.4	69.7	N/A
Canine^b^	10/Te/07	N/A	N/A	76.4/87.4	64.2	N/A	N/A	N/A	75.8/86.9	66.0	N/A
Vaccine	255	79.1	79.5/92.4	77.7/86.4	69.4	82.6/89.7	78.9	79.6/91.9	76.4/87.0	72.4	84.7/94.4

N/A represents not applicable; ^a^ the complete ORF2 gene sequence of the 213/95 strain isolated from a dog with diarrhea was obtained from GenBank database (AF283778); ^b^ the partial ORF2 gene sequence of the 10/Te/07 strain isolated from a dog with gastroenteritis was obtained from GenBank database (EU980610); ^c^ the identity of E region of VP1 protein between different strains at the amino acid level; R means respiratory origin; E means enteric origin. The FCV strain 255 is the commercial vaccine widely used in China.

**Table 3:** Comparative studies on susceptibility to acid, trypsin and bile salts between FCVs from dogs and cats.

Host	FCV strain	Viral titers (log10 TCID50) and log10 reduction resulted from the treatment indicated						
		Control	HCl (pH 3.0)		Trypsin (0.5%)		Bile (0.5%)	
			Treated	Δ	Treated	Δ	Treated	Δ
Canine	GXHC01-21	7.22	0	7.22	5.61	1.61	6.83	0.39
Canine	GXNN10-21	6.30	5.06	1.24	3.67	2.63	4.47	1.83
Feline (respiratory)	GX01-13	7.11	4.22	2.89	4.38	2.73	6.50	0.61
Feline (enteric)	GXNN04-20	6.83	0	6.83	5.60	1.23	6.67	0.16

Abbreviation: Δ, log_10_ reduction.

## Data Availability

The data that support the findings of this study are available from the corresponding author upon reasonable request.

## Supplementary Materials

Supplementary Materials Table S1: genomic information of two canine-origin FCVs.
