# Supplementary material for: Genetic Evolution and Biological Characteristics of Feline Caliciviruses Isolated from Dogs
Source: Transbound Emerg Dis. 2023 Feb 27;2023:1145176. doi: 10.1155/2023/1145176 (PMC12017032; doi:10.1155/2023/1145176)
Supplement: Supplementary Materials — Table S1: genomic information of two canine-origin FCVs. [file 1145176.f1.doc]

| Host | Strain | Country | Year | Samples | 5′UTR | ORF1 | ORF2 | ORF3 | 3′UTR | Total genome Length |
| --- | --- | --- | --- | --- | --- | --- | --- | --- | --- | --- |
| Canine | GXHC01-21 | China: Guangxi | 2021 | fecal | 1-19 | 20-5311  (5292 nt/1763 aa) | 5314-7323  (2010 nt/669 aa) | 7320-7640  (321 nt/106 aa) | 7641-7721  (81 nt) | 7721 nt |
| Canine | GXNN10-21 | China: Guangxi | 2021 | feces | 1-19 | 20-5311  (5292 nt/1763 aa) | 5314-7323  (2010 nt/669 aa) | 7320-7640  (321 nt/106 aa) | 7641-7722  (82 nt) | 7722 nt |

**TABLE S1 Genomic information of two canine-origin FCVs**
